# Carbonic Anhydrase-8 Regulates Inflammatory Pain by Inhibiting the ITPR1-Cytosolic Free Calcium Pathway

**DOI:** 10.1371/journal.pone.0118273

**Published:** 2015-03-03

**Authors:** Gerald Z. Zhuang, Benjamin Keeler, Jeff Grant, Laura Bianchi, Eugene S. Fu, Yan Ping Zhang, Diana M. Erasso, Jian-Guo Cui, Tim Wiltshire, Qiongzhen Li, Shuanglin Hao, Konstantinos D. Sarantopoulos, Keith Candiotti, Sarah M. Wishnek, Shad B. Smith, William Maixner, Luda Diatchenko, Eden R. Martin, Roy C. Levitt

**Affiliations:** 1 Department of Anesthesiology, Perioperative Medicine and Pain Management, University of Miami Miller School of Medicine, Miami, Florida, United States of America; 2 Department of Physiology and Biophysics, University of Miami Miller School of Medicine, Miami, Florida, United States of America; 3 Department of Pharmacology and Experimental Therapeutics, Eshelman School of Pharmacy, University of North Carolina, Chapel Hill, North Carolina, United States of America; 4 Algynomics Inc., Chapel Hill, North Carolina, United States of America; 5 John P. Hussman Institute for Human Genomics, University of Miami Miller School of Medicine, Miami, Florida, United States of America; 6 John T Macdonald Foundation Department of Human Genetics, University of Miami Miller School of Medicine, Miami, Florida, United States of America; 7 Bruce W. Carter Miami Veterans Healthcare System, Miami, Florida, United States of America; University of Sao Paulo, BRAZIL

## Abstract

Calcium dysregulation is causally linked with various forms of neuropathology including seizure disorders, multiple sclerosis, Huntington’s disease, Alzheimer’s, spinal cerebellar ataxia (SCA) and chronic pain. Carbonic anhydrase-8 (Car8) is an allosteric inhibitor of inositol trisphosphate receptor-1 (ITPR1), which regulates intracellular calcium release fundamental to critical cellular functions including neuronal excitability, neurite outgrowth, neurotransmitter release, mitochondrial energy production and cell fate. In this report we test the hypothesis that Car8 regulation of ITPR1 and cytoplasmic free calcium release is critical to nociception and pain behaviors. We show Car8 null mutant mice (MT) exhibit mechanical allodynia and thermal hyperalgesia. Dorsal root ganglia (DRG) from MT also demonstrate increased steady-state ITPR1 phosphorylation (pITPR1) and cytoplasmic free calcium release. Overexpression of Car8 wildtype protein in MT nociceptors complements Car8 deficiency, down regulates pITPR1 and abolishes thermal and mechanical hypersensitivity. We also show that Car8 nociceptor overexpression alleviates chronic inflammatory pain. Finally, inflammation results in downregulation of DRG Car8 that is associated with increased pITPR1 expression relative to ITPR1, suggesting a possible mechanism of acute hypersensitivity. Our findings indicate Car8 regulates the ITPR1-cytosolic free calcium pathway that is critical to nociception, inflammatory pain and possibly other neuropathological states. Car8 and ITPR1 represent new therapeutic targets for chronic pain.

## Introduction

Chronic inflammation disrupts calcium-homeostasis [1] within the endoplasmic reticulum (ER), which is causally linked to various forms of neuropathology including spinal cerebellar ataxia (SCA), seizure disorders, multiple sclerosis, Huntington’s disease, Alzheimer’s and chronic pain [2]. Stimulus-triggered calcium release from ER calcium stores represents one of the most ubiquitous signaling systems in biology [3]. Tightly controlled calcium release channels and pumps residing in the ER membranes regulate many critical cellular functions including synaptic plasticity underlying long-term potentiation and persistent pain [4–9]. Elevated cytosolic calcium was also shown to contribute to chronic pain through enhanced mitochondrial calcium uptake, and the increased production of reactive oxygen species [10]. These calcium release functions are maintained by inositol trisphosphate receptors (ITPRs) and ryanodine receptors [11, 12]. In particular, ITPRs are thought to function as “coincidence detectors” to transduce concurrent signals resulting from activation of metabotropic receptors producing inositol 1,4,5-trisphosphate (IP3) ligand and cellular entry of calcium through voltage-gated and receptor-gated calcium channels (such as N-methyl-D-aspartate receptors) [13–15], which have been shown to play an important role in chronic pain behaviors [16]. Despite its central role in neuronal functioning and neuropathology [17, 18], little is known about ITPR dysregulation in pain and pain-related behaviors.

ITPR1 is the major neuronal IP3 receptor subtype and contains five functionally distinct domains [19], 1) the IP3 ligand-binding core and ‘suppressor’ domain near the N-terminus [20, 21]; 2) the ‘modulatory’ domain responding to intracellular modulators such as calcium, calmodulin, ATP, carbonic anhydrase-8 (Car8) [22, 23] and phosphorylation by several protein kinases [24–26]; 3) a region containing six transmembrane domains; 4) a ‘gatekeeper’ domain [3]; and 5) a cytoplasmic C-terminal tail which interacts with numerous regulatory proteins [27–30]. While IP3 and calcium are important co-regulators of ITPR1 [31], this channel also has two PKA (cAMP-dependent protein kinase) consensus sequences at Ser-1589 and Ser-1755 that can be phosphorylated in response to cAMP accumulation [32]. Through alternative splicing, the neuronal form of ITPR1 (long form) retains a 40 amino acid segment that is activated primarily through phosphorylation at Ser-1755, and this phosphoregulation has dramatic effects on calcium release [24, 32–41]. Moreover, ITPR1 dependent increases in intracellular calcium concentration can activate various effectors, including protein kinase C (PKC) and calcium/calmodulin-dependent kinase (CaMK) that are important to the initiation of persistent pain [42–44]. In addition, early work suggests that PKC may also mediate persistent pain by depolarizing unmyelinated afferent neurons[45], and sensitizing afferent neurons [46, 47]. Further, nuclear free calcium was shown to integrate synapse-to-nucleus communications thereby regulating ‘spinal genomic responses’ required for persistent pain [48].

Car8 belongs to a family of regulatory proteins that impact ITPR1 function [27–30]. Unlike most members of the carbonic anhydrase super gene family, Car8 lacks enzymatic activity to hydrate CO_2_ due to the absence of zinc coordinating histidine residues within the active site [49]. Instead, Car8 functions as an allosteric regulator of the ITPR1 intracellular calcium release channel by altering the affinity of ITPR1 for the IP3 ligand, resulting in the modulation of excitatory calcium signaling [22, 23]. Causative mutations in ITPR1 and its Car8 regulatory protein are both causally linked to SCA disorders in mice and humans [50]. SCA is a rare genetic disorder frequently associated with debilitating problems in mobility, self-care, depression, anxiety, and pain [51]. The SCA *waddle* (*wdl*) mouse, which has a spontaneous null mutation of Car8, exhibits gait ataxia and dystonia. The *wdl* mouse has no gross morphological abnormalities in the cerebellum, however alterations have been observed at synapses between Purkinje cell dendritic spines and parallel fiber varicosities [23], suggesting a role for Car8 in synaptic formation and / or maintenance also likely relevant to persistent pain.

While these important sensory neuron functions implicate Car8 as a candidate gene, to date there have been no investigations on the impact of Car8 deficiency on its role in pain. Herein, to address these important questions, we undertook a series of studies to test the hypothesis that Car8 is critical to the regulation of ITPR1, cytosolic free calcium release and homeostasis, and pain related behaviors. Using *in vitro* and *in vivo* techniques, we show that Car8 *wdl* null mutant mice (MT) demonstrate thermal and mechanical hypersensitivity at baseline; and are susceptible to inflammatory pain related behaviors. We further show that Car8 functions to inhibit modulatory domain phosphorylation of murine ITPR1 and intracellular calcium release. Moreover, we demonstrate that overexpression of the Car8 wildtype protein in nociceptors after gene transfer via sciatic nerve injections into MT mice down regulates pITPR1 at Ser-1755, decreases steady-state cytoplasmic free calcium, inhibits ATP-stimulated calcium release, and abolishes mechanical allodynia and thermal hyperalgesia. In addition, we demonstrate inflammation-induced hyperalgesia and a relative reduction of Car8 protein to ITPR1 expression as a potential mechanism of inflammatory pain that was reversed by overexpression of the Car8 wildtype protein after gene transfer in mice. These discoveries establish a critical role for Car8 in nociception and complex inflammatory pain-related behaviors that may facilitate the development of novel targeted pain therapies.

## Materials and Methods

### Animal preparations and care

Car8 deficient SCA mice are derived from C57BLKS/J due to a 19 Bp deletion within exon 8 of the Car8 gene. Car8 wild type (C57BLKS/J, WT), Car8 heterozygous (HET) and Car8 homozygous (MT) mice are obtained from Jackson Laboratory (Bar Harbor, ME). MT mice display waddling and ataxic motor behavior while WT and HET mice demonstrate no waddling and ataxia phenotypes. MT mice were generated through cross breeding of HET and MT mice. MT mice can also be easily distinguished from HET mice according to their genotypes. The PureLink Genomic DNA Mini Kit (Invitrogen, K1820-01) was used to extract genomic DNA following the protocol provided by the company to check for the 19 Bp deletion. PCR was performed in the 2720 thermal cycler of Applied Biosystems (Invitrogen) using primers described below (Generation of Vectors and RT-PCR sections). The PCR segment amplified includes the 19-bp deletion in exon 8 on chromosome 8. The 19 Bp difference between WT and MT mice was distinguished by running PCR products in mini-PROTEAN TBE Precast Gels (Bio-Rad, Catalog # 456-5055). All procedures related to animal use and care, were pre-approved by the University of Miami Institutional Animal Use and Care Committee. All mice were males of 2–3 months of age, weighing 20–35 grams; all rats Sprague Dawley rats (2–4 months of age). All animals were housed in a 12 h. light/dark cycle in a virus/antigen-free facility with controlled temperature and humidity and were provided with water and food *ad libitum*.

### Pain behavioral testing

Behavioral tests were conducted in a quiet room maintained at 23–25°C. Familiarization with each testing paradigm was achieved for all mice with repeated measurements for at least 60 min for at least 5 consecutive days before collecting baseline and test measurements. Behavioral tests were performed in a blinded fashion. For testing mechanical sensitivity, animals were put under inverted round plastic box (Radius: 9 cm, Height: 11 cm) on an elevated mesh floor. The hind paw was pressed with one of a series of von Frey filaments with logarithmically incrementing stiffness (0.16, 0.4, 0.6, 1, 1.4, 2, 4, 6) (Stoelting Co., Wood Dale, IL) presented perpendicular to the plantar surface of each hind paw for 1–2 s with an inter-stimulus interval of at least 5 s. The 50% threshold was determined using Dixon’s up-down method [52]. Briefly, ascending or descending stimuli were applied in a consecutive fashion starting with the 1.0 g monofilament that was normally smaller than the mouse baseline threshold. In the absence of a paw withdrawal to a filament, a stronger stimulus was applied whereas in the presence of a paw withdrawal, the next weaker stimulus was applied. The resulting pattern of positive and negative responses was tabulated using the convention: X = withdrawal; O = no withdrawal and the 50% response threshold was interpolated using the following formula: 50% threshold = (10^[Xf+κδ])^/10,000) [53]. For testing thermal sensitivity, animals were put in a plastic box placed on a glass plate, and the plantar surface was exposed to a beam of radiant heat through a transparent Perspex surface [54, 55]. The baseline latencies were adjusted to 5–9 sec with a maximum of 15 sec as cutoff to prevent potential injury. The latencies were averaged over three trials, separated by a 5 min interval.

### Generation of AAV vectors expressing wildtype or mutant Car8 with V5 tag

Car8 wild type (WT) gene cDNA was purchased from ATCC (Cat. No. 4216498). WT gene was amplified by Eppendorf Recycler gradient (Model 5331) and cloned between the BamHI and XhoI (NEB) restriction sites of the pcDNA3.1/V5-His A (Invitrogen Life Technologies, Carlsbad, CA) using the forward primer: TTTGGATCCGCCACCAT- GGCTGACCTGAGCTTCATTG and the reverse primer: TTTCTCGAGCTGAAAGGCC- GCTCGGATG. The V5-Car8 construct was then amplified from pcDNA3.1/V5-His A and cloned between the BamHI and BglII restriction sites of the pAAV-MCS vector, one component of AAV Helper-Free System (Agilent Technologies, Santa Clara, CA) using the forward primer: CTCGGATCCGCCACCATGGC and the reverse primer: CTCGGA- TCCGCCACCATGGC. The cDNA of Car8 mutant (MT) with deletion of 19 bp in exon 8 was made by using the GENEART Site-Directed Mutagenesis System (Invitrogen Life Technologies, Carlsbad, CA). Because the system can just delete less than 12 bp at one time we used 2 steps to delete 19 bp. The 1st step deleted 10 bp AAGGCTGAGG from 768 to 777 in the CDS sequence using the forward primer: ATG- CAGATAGAAGAATTTCGACACATGTCAAGGGGGCAGA and the reverse primer: TC- TGCCCCCTTGACATGTGTCGAAATTCTTCTATCTGCAT. The 2^nd^ step deleted 9 Bps ACACATGTC from 778 to 786 using the forward primer: TGCAGATAGAAGAATTTCG- AAGGGGGCAGAACTGGTGGAG and the reverse primer: CTCCACCAGTTCTGCCC- CCTTCGAAATTCTTCTATCTGCA. After introducing the 19 bp deletion of Car8 (Car8^MT^) in pcDNA3.1/V5-His A, the reading frame shifted generating a new stop code and premature termination. Using PCR to copy the Car8^MT^ in the new reading frame the amplified product was cloned between the BamHI and XhoI restriction sites of pcDNA3.1/V5-His A using the forward primer: TTTGGATCCGCCACCATGGCT and the reverse primer: TTTCTCGAGGGGGCTGGGTAGGTCGGAAAT. The V5-Car8^MT^ construct was amplified from pcDNA3.1/V5-His A and cloned between the BamHI and BglII restriction sites of the pAAV-MCS vector using the forward primer: CTCGGATCC- GCCACCATGGC and the reverse primer: TTTAGATCTTCACGTAGAATCGAGACCG-AGGAGAG. The recombinant AAV8-V5-Car8 viral particles were produced by the Miami Project Viral Vector Core, University of Miami Miller School of Medicine. Briefly, the vector plasmids, and the packaging plasmid AAV8 733(5) and pHelper (Agilent Technologies, Santa Clara, CA) were co-transfected into HEK293 cells at 70% confluence using calcium phosphate precipitation method. The cells were incubated for 48 hours at 37°C and 5% CO_2_. After 48 hours, the cells were collected and freeze-thawed three times to release the AAV particles from the cells. After 30 min of Benzonase Nuclease (Sigma) treatment, the crude lysate is clarified by low speed centrifugation. The supernatant was loaded on discontinuous iodixanol step gradients(6) in OptiSeal tubes (Beckman Coulter) and centrifuged in a Type 70 Ti rotor (Beckman Coulter) at 69,000 rpm (350,000g) for 1 h at 18°C. The AAV particles containing section was collected and further purified using an AKTA FPLC system (GE Healthcare) by column chromatography on a 5 ml HiTrap column (GE Healthcare). About 25 mL was eluted from the column using elution buffer (20 mM Tris, 215 mM NaCl, pH 8.0) and then the AAV particles were concentrated and buffer exchanged to 200μl in HBSS (Invitrogen) using an Amicon Ultra-15 50K concentrator (Millipore). The purified AAV particles were then titrated for genome contents using real-time qPCR method. Usually the titers were in the range 1–3 x 10^14^ GC (Genome Copy) per mL.

### Sciatic nerve injections of AAV8-V5-Car8 viral particles

Male mice were anesthetized by intraperitoneal injection of ketamine, xylazine and acepromazine (VEDCO, Saint Joseph, Mo). After sciatic nerve exposure, 1.5 μl viral particles of 1.3E+14 *AAV8-V5-Car8* ^*WT*^, 1.6E+14 *AAV8-V5-Car8* ^*MT*^ and 1.7E+12 *AAV2-eGFP* were injected into the sciatic nerve through a 35-gauge NanoFil needle using a NanoFil syringe (World Precision Instruments, Sarasota, FL). The injection site was approximately 45 mm from the tip of the third toe. The needle remained at the injection site for 1 additional min before it was slowly removed.

### Tissue and cell culture preparation

Mice and rats were anesthetized with isoflurane or a ketamine, xylazine and acepromazine cocktail, respectively, and perfused through the left ventricle with saline (30mL) followed by 4% paraformaldehyde with 15% saturated picric acid solution in 0.16 M PBS (pH 7.2–7.4, 4°C). Spinal cord and dorsal root ganglion (DRG) were dissected, and put in the same fixative for 2–4 h post fix, then transferred to 20% sucrose overnight or until the tissue touched the bottom. The tissue was embedded with OCT (Andwin Scientific Inc, Schaumburg, IL) on dry ice. 16 μm sections were cut by Leica 1900 Cryostat (Leica Microsystems Inc., Buffalo Grove, IL) and mounted to slides for immunofluorescence staining. Fresh spinal cord and DRG were dissected directly from mice or rats for western blot. HEK293 and Neuro-2a (N2A, ATCC) cells were plated in 24-well plates on poly-D-lysine-, laminin-coated glass coverslips for immunocytochemistry and calcium imaging or in 6-well plates for protein collection and western blot. Cells were seeded at a density of 2 x 10^5^ cells/ml. Culture volume was 2 mL per well in 6-well plates, 0.5 ml per well in 24-well plates. The cultures were incubated at 37°C in a water saturated atmosphere containing 5% CO_2_/95% air and maintained in Gibco DMEM (Invitrogen) supplemented with 10% FBS (Invitrogen) and 1X cellgro penicillin-streptomycin (Fisher Scientific, Pittsburg, PA).

### Transfections

HEK293 and N2A cells were plated at 4 x 10^5^ cells for 6-well plates and 1 x 10^5^ cells for 24 well plates to obtain a 90% confluent layer after 24 hours. Transfection was performed using Lipofectamine 2000 according to the manufacturer’s instructions. Briefly, transfections were performed in Opti-MEM I Reduced Serum Medium (Invitrogen), using 2 μg DNA and 6 μl Lipofectamine 2000 for 6-well plates or 0.5 μg and 2 μl for 24-well. Cells were maintained in transfection media for 4–6 h, and then transfection media was replaced with DMEM plus FBS and penicillin-streptomycin. After 48 h incubation, cells were used for measurements of mRNA and protein expression using real-time PCR, immunocytochemistry and western blot.

### Immunocytochemistry and imaging

The immunostaining was performed as described previously [56]. Briefly, brain and spinal cord tissue sections and cell cultures were fixed by 4% PFA in PBS for 30 min, permeabilized in 0.3% Triton X-100 for 1.5 h at room temperature, and blocked in 4% normal serum (Jackson ImmunoResearch Laboratories, West Grove, PA) for 20 min. Primary antibodies specific for CA8, V5 (Abcam, Cambridge, MA), pITPR1 (Ser-1755), ITPR1, β-tubulin and β-actin (Cell Signaling Technology, Danvers, MA), Tuj1 (Covance, Princeton, NJ), S100 (DAKO, Carpinteria, CA), Substance P (SP, BD Biosciences, San Jose, CA), calcium gene-related peptide (CGRP), isolectin B4, biotin conjugate (IB4-Biotin, Sigma), and neurofilament 200 (NF200, Sigma, St. Louis, MO) were diluted in PBS containing 0.1% Triton X-100 and allowed to incubate with sections and cell cultures overnight at 4°C. Sections and cultures were washed three times for 10 min each in PBS, and incubated with Alexa Fluor 488 and/or Alexa Fluor 594-conjugated second antibodies (Invitrogen) for 1h at room temperature. Sections and cultures were washed again in PBS and cover slipped using Gel/Mount anti-fading mounting media (Biomeda, Foster City, CA). DAPI (Sigma) was mixed with 2^nd^ antibody for total cells number counts. Images were acquired using an inverted microscope (DMI 6000 B, Leica, Germany). Tuj1 staining was used for total neuron counting. Gamma, gain and exposure levels were set for control sections and kept constant for all other sections within an experiment. ImageJ 1.45 was used to measure positive cell body areas and immunostaining intensity.

### Western blot assays

DRGs, HEK293 and N2A cultures were homogenized in RIPA buffer with a mixture of proteinase and phosphatase inhibitors (Sigma). Protein samples were generally separated on 4–15% SDS polyacrylamide gels and transferred to a nitrocellulose membrane. For large proteins, like pITPR1 western blot, 5% Tris-HCl gel and 10% methanol in transfer buffer were used to increase efficiency. The blots were blocked with 5% milk in Tris-buffered saline (TBS) with 0.1% Tween 20 for 1 h at room temperature and incubated overnight at 4°C with primary antibodies. The blots were then incubated for 1 h at room temperature with HRP-conjugated secondary antibodies (Jackson ImmunoResearch Laboratories) and bands were visualized using Pierce SuperSignal substrate (Thermal Scientific, Rockford, IL). The following primary antibodies were used: anti-Car8 (Santa Crutz, Santa Crutz, CA), anti-V5 (Invitrogen), anti-pITPR1 (Cell Signaling Technology, Ser-1755), anti-β-tubulin and anti-β-actin (Sigma). Density analysis was performed using UN-SCAN-IT, standardized to β-actin, and a one-way ANOVA was used for statistical analysis.

### RNA extraction, reverse transcription-polymerase chain reaction (RT-PCR) and real time PCR (qPCR)

Total RNA was extracted from cultured N2A cells and DRG tissue using the TRIzol reagent (Invitrogen) following the manufacturer’s protocol. Total RNA was quantified by an Epoch spectrophotometer (BIOTEK). Two-step RT-PCR was performed using the ImProm-IITM Reverse Transcription System and GoTaq Flexi DNA Polymerase (Promega, Madison, WI) according to the supplier’s protocols. For some RNA samples, RNA was treated by AMPD1 DNase (SIGMA-ALDRICH) to remove genomic DNA before reverse transcription. RT-PCR was used to amplify endogenous Car8 with the forward primer: GCTTGAAGGCTGTGACTGAG and the reverse primer: ATGTGTCCTCAGCCTTCGAA. The PCR products were loaded on 1% agarose gel, and were visualized with ethidium bromide. No template was used as negative control and the cerebellum from adult Car8^WT^ mice as positive control. Both negative control and positive control were applied in each running. qPCR was used to amplify exogenous Car8 with the forward primer: CCTTGCAGCGAAGGAGTTAC and the reverse primer: GGAGAGGGTTA GGGATAGGC. GAPDH was used as internal control with the forward primer: CAACTCCCACTCTTCCACCT and the reverse primer: CTTGCTCAGTGTCCTTGCTG. qPCR and analysis was carried out on a StepOne Plus system (Applied Biosystems, Invitrogen) using Power SYBR green PCR master mix (Applied Biosystems). Primers (final concentration = 250 nM) were designed across the border of the carbonic anhydrase sequence and the V5 tag in the vector to ensure endogenous Car8 was not amplified and after the mutation to ensure both WT and mutant versions would amplify similarly. Car8 results were normalized to the expression of GAPDH respectively. Efficiency of each primer pair was determined via standard curve and used to determine relative quantities of starting mRNA amounts.

### Calcium assay methods

HEK293 cells were transfected with AAV2-eGFP, AAV2-V5-Car8^WT^ or AAV2-V5-Car8^MT^ vectors 48 hours prior to assaying according as described previously. 24 hours before the assay, 100,000 cells were then split onto 12 mm glass coverslips (Propper, Long Island, NY) coated with poly-lysine overnight and laminin for 1.5 h. Primary DRG cultures were plated directly onto the poly-lysine and laminin coated coverslips and allowed to acclimate for 48 hours before being treated with 20 μM camptothecin for 48 hours to remove non-neuronal cells [57]. On the day of the assay, media was replaced with dye from the Fluo-4 NW Calcium Assay Kit (Life Technologies) and cells were incubated at 37°C for 30 minutes and then room temperature for an additional 30 minutes per the manufacturer’s specifications. Coverslips were loaded onto an upright microscope and perfused with Ca^2+^-free media (all concentrations in mM: 130 NaCl; 4.7 KCl; 2.3 MgSO_4_; 5 Glucose; 20 HEPES; 10 EGTA; 1.2 KH_2_PO_4_, pH 7.4) as described by Boehmerle et al., 2007 [58]. Cells were allowed to equilibrate for 5 minutes before recording on T.I.L.L. Photonics GMBH Imaging System II (Photonics, Germany) was initiated. Cells were visualized every second over 10 minutes while media alone or media containing ATP was perfused onto cells in Calcium-free media. Individual cells were analyzed using ImageJ to measure fluorescent intensity.

### Quantification and statistics

To quantify immunoreactive staining of Car8, pITPR1 and V5 expression in the DRG, the percentages of positive neurons in the L5 and L4 DRG from four nonadjacent sections were determined as described previously [59]. Briefly, the DRGs were serially sectioned at 16 μm. The total number of positive neurons was divided by the total number of Tuj1 positive neurons in the DRG sections, and percentage of positive neurons was calculated. Percentages were calculated from 3–4 sections from one animal and were averaged as the percentage for that animal. Three or 4 mice were used for analysis of each group. To quantify immunoreactive double-staining in the DRG, the total number of markers including CGRP, SP, IB4, S100 and NF200 positive neurons was divided by the total number of positive V5 neurons and percentage was calculated. The person making the quantitative evaluation was blinded to the group and the arrangement of the ganglia on the slides. Data were presented as mean ± SEM. Differences between groups were compared using Student’s t-test, or ANOVA, followed by Fisher’s PLSD test. The criterion for statistical significance was P < 0.05.

## Results

### Car8 deficiency produces mechanical and thermal hypersensitivity

**Fig 1:**
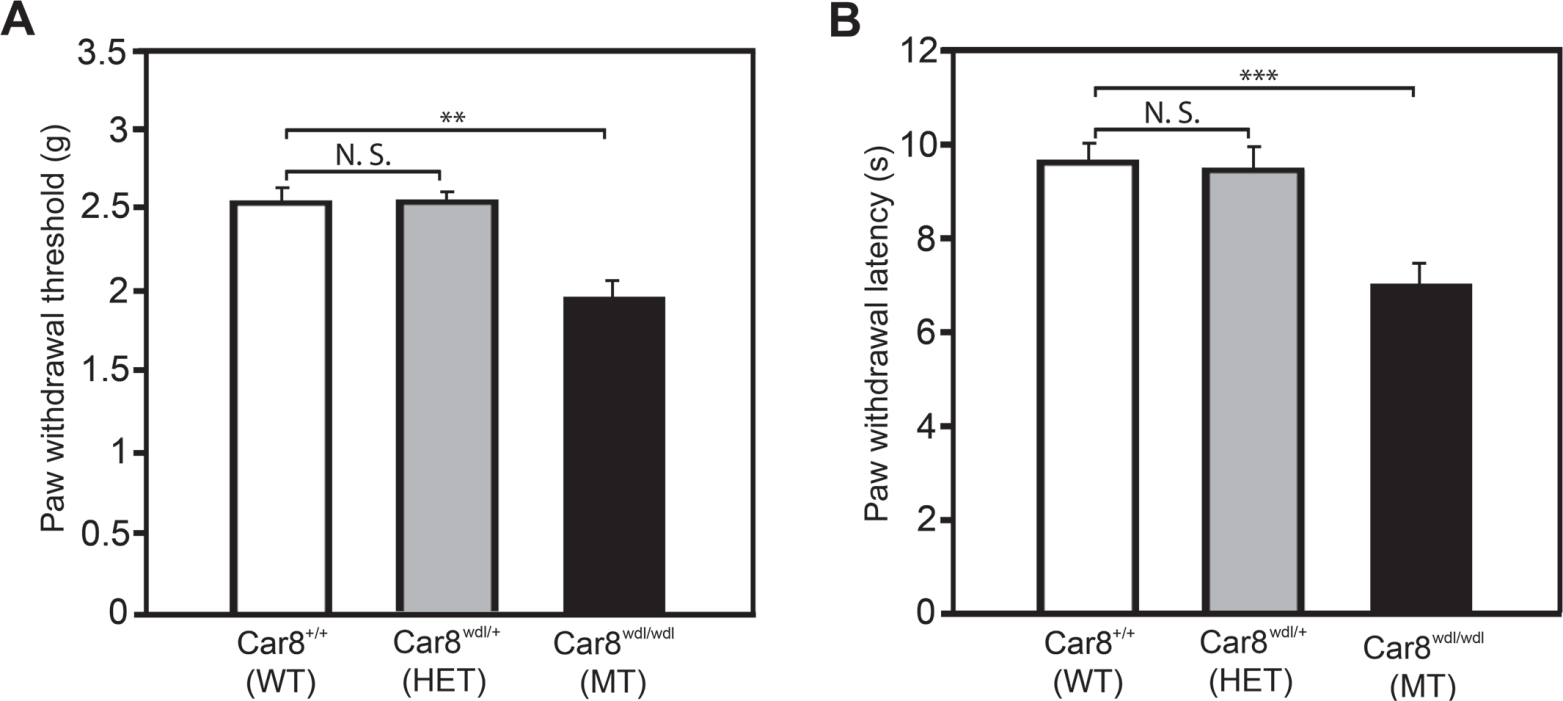
Car8 deficiency alters nociception by inducing hypersensitivity. Nociception was tested in background C57BLKS/J (WT) mice (white bars), C57BLKS mice heterozygous for a 19 Bp deletion in exon 8 of the *Car8* gene (*Car8 wdl+/−*)(HET) (grey bars); and C57BLKS mice homozygous for this deletion *(Car8 wdl−/−*)(MT) (black bars). (A) The “up-down” method (see Methods for details) was used to measure mechanical responses by probing the plantar aspect of the hindpaw with von Frey filaments and determining the paw withdrawal threshold (grams). (B) Thermal withdrawal response latencies were measured to radiant heat (75 units) applied to the plantar aspect of the hind paw (seconds). (N = 12; ** P<0.01; *** P<0.001; one way ANOVA)

To address the role of Car8 in nociceptor functioning, we tested mechanical and thermal sensitivity in naïve C57BLKS wildtype mice (WT); C57BLKS mice homozygous for the 19-base pair (BP) deletion in exon 8 (*Car8 wdl*−/−)(MT); and C57BLKS mice heterozygous at the *Car8 wdl*+/− locus (HET). This “*waddles*” mouse (*wdl*) harbors a deletion that destabilizes the Car8 protein leading to nearly complete absence of this protein in nervous tissues of MT mice that display wobbly side-to-side ataxic movements [23, 60]. The *wdl* mouse demonstrates no gross morphological abnormalities in the cerebellum and resting fore limb and hind limb tone is normal [60], suggesting these mice are suitable for pain behavior testing. Both mechanical withdrawal threshold (P<0.01; Fig. 1A) and thermal withdrawal latency (P<0.001; Fig. 1B) are significantly decreased in MT mice as compared to HET and WT mice. Our neurobehavioral data demonstrate that Car8 deficiency causes both mechanical allodynia and thermal hyperalgesia at baseline. Because HET mice failed to display an abnormal neurobehavioral phenotype, forthcoming experiments focused on comparisons between WT and MT mice.

### Car8 protein is highly expressed in sensory neurons and deficient in MT mice

**Fig 2:**
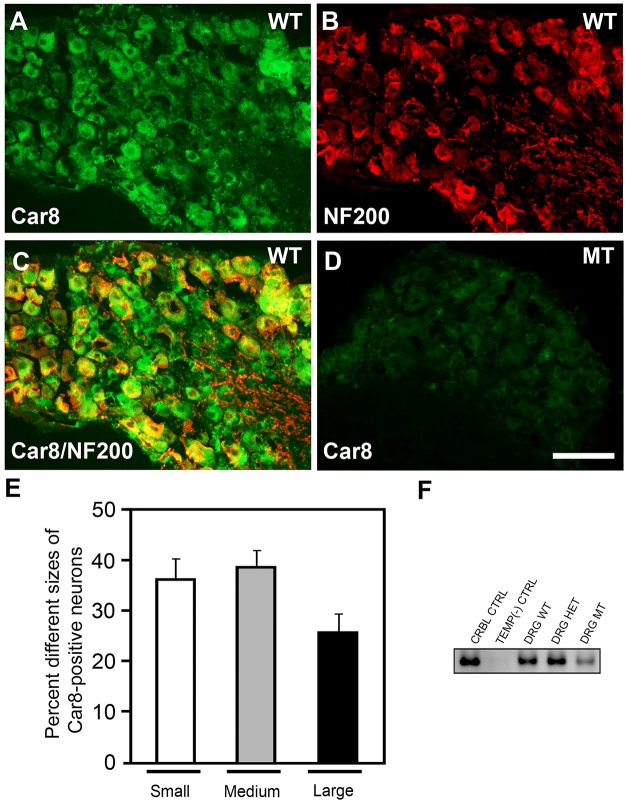
DRG Car8 expression WT and MT animals. (Fig. 2A-D) Immunoreactivity for anti-Car8 (Fig. 2A, D, green), anti-NF200 (Fig. 2B, red), and anti-Car8 with anti-NF200 (Fig. 2C) antibodies, respectively. The merged image (Fig. 2C) is from A and B. Immunohistochemistry demonstrates Car8 is expressed in the WT DRG (Fig. 2A) but little or none in the MT DRG (Fig. 2D). Percentage of different size neurons of Car8-containing neurons (measuring neuronal somata with visible nuclei) in the WT DRG (Fig. 2E). Small: <300 μm^2^; Medium: 300–700 μm^2^; Large: >700 μm^2^. RT-PCR demonstrates *Car8* mRNA in DRG tissues (Fig. 2F). No template (TEMP) was used as a negative control (CTRL). The cerebellum was used as positive control. N = 4. Scale bar: 100 μm.

Little is known about Car8 expression in the peripheral nervous system of mice. Using immunohistochemistry (IHC), we next tested if Car8 was expressed in DRG neurons of these mice. In Fig. 2 we show DRG immunostaining of Car8 in these wildtype mice and Car8 deficient mice. As expected, IHC shows all DRG neurons in MT mice are deficient in Car8 protein as compared to the WT mice. We also examined *Car8* mRNA expression in DRG from WT mice using PCR (Fig. 2F). Positive controls used RNA from adult cerebellum (CRBL), while RT-PCR not containing template was used as a negative control. In contrast to Car8 protein, at steady state we observed mRNA in DRG from WT, HET and MT mice. Collectively, these neurobehavioral and neuroanatomical data from DRG of WT and MT mice suggest Car8 may have an important role in nociceptor functions.

Primary sensory neurons in WT mice represent a diverse cell population. We classified neuronal somata with a visible nucleus by size as follows [61]: small C-fiber and polymodal nociceptive neurons <300 μm^2^, Aδ thermal and mechanical nociceptive neurons denoted as medium-sized 300–700 μm^2^, and large Aβ proprioceptive neurons >700 μm^2^. To determine the size distribution of (μm^2^) Car8-positive cells in relation to total DRG neurons in WT mice, we used double staining with Tuj1 (immunoreactive data not shown). Seventy-four percent of Car8-positive neurons corresponded to small to medium-sized cells (<700 μm^2^). Double immunofluorescence staining of Car8 and neurofilament 200 (NF200), a marker of A-fiber neurons [62] was performed to further characterize the tropism of Car8-positive DRG neurons. While 33% of Car8 neurons co-localized with NF200 (Fig. 2A-C, E) suggesting Car8-positive DRG cells are primarily in small to medium nociceptive neurons, Car8 deficiency in larger DRG neurons may also contribute to sensory motor dysfunction occurring at this level of the neuraxis.

### Steady state DRG intracellular pITPR1 and free calcium levels are increased by Car8 deficiency

ITPR1 plays a crucial role in a variety of cell functions by converting IP3 signaling into calcium signaling [63, 64]. It has been shown that protein kinase A (PKA) activation by forskolin increases cyclic adenosine monophosphate (cAMP) to phosphorylate ITPR1 [65]. ITPR1 phosphorylation increases channel activity in planar lipid bilayers, regulating calcium-dependent signaling [46, 66, 67]. Car8 binds to ITPR1 and reduces the affinity of the receptor for the IP3 ligand, which reduces IP3-induced calcium release in Purkinje cells [22]. We hypothesized that MT mice with Car8 deficiency associated with increased pain related behaviors exhibit elevated ITPR1 phosphorylation associated with activation and increases in cytosolic free calcium.

**Fig 3:**
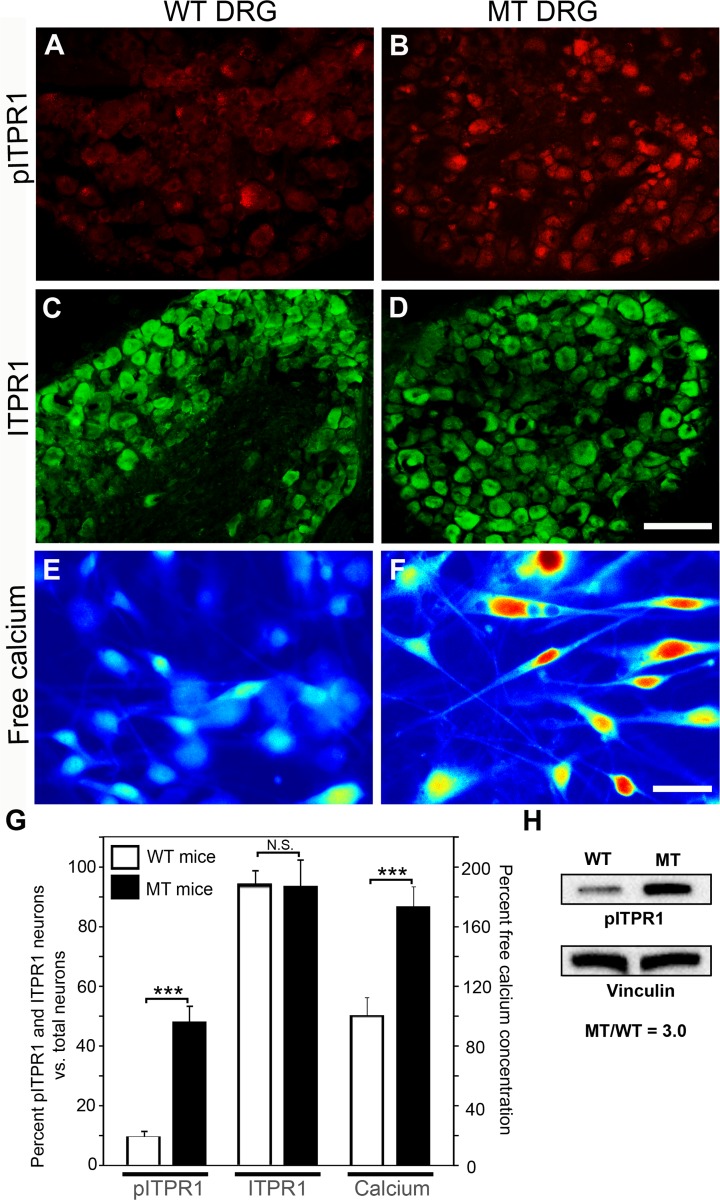
DRG ITPR1, pITPR1 and steady-state cytoplasmic free calcium in WT and MT mice. Immunohistochemistry data show there are no significant differences in ITPR1 DRG expression between WT and MT mice (Fig. 3C, D); pITPR1 was higher in MT DRG as compared to WT DRG (Fig. 3A, B, G). Western blot data also show an increase of pITPR1 in DRG from MT mice as compared to WT mice (ratio MT/WT pITPR1 = 3.0)(Fig. 3H). Vinculin was used as a loading control. Calcium image analyses from cultured DRG cells also demonstrate free calcium concentration is higher in MT DRG as compared to WT DRG (Fig. 3E, F, G) N = 4. Scale bar: 100 μm (Fig. 3A-D), 30 μm (Fig. 3E-F). (*** P<0.001; Student’s *t*-test or one-way ANOVA.)

Here we show that while steady-state ITPR1 expression does not differ at baseline in DRG neurons between WT and MT mice (Fig. 3C and 3D), Car8 deficiency is associated with increased pITPR1-positive DRG neurons in MT mice as compared to WT mice (Fig. 3B, MT mice 60.1% vs. Fig. 3A, WT mice 11.6%, P = 0.001) (summarized in Fig. 3G). Western blotting from MT and WT DRG corroborate these findings (Fig. 3H). Collectively, these data show that the mechanical allodynia and thermal hyperalgesia exhibited by MT mice (see Fig. 1) are associated with increased steady-state DRG pITPR1 (Fig. 3). To further examine whether the hypersensitivity is associated with a difference in baseline steady-state cytoplasmic free calcium, we cultured adult DRG neurons from WT and MT mice. Steady-state cytoplasmic free calcium in naïve animals was clearly higher in DRG from MT mice compared with WT mice (Fig. 3E-G) consistent with a lack of Car8 protein and increased steady-state activation of ITPR1. Overall, these data suggest Car8 deficiency in sensory neurons in MT mice is associated with increased pITPR1 and higher steady-state cytoplasmic free calcium levels, which are associated with thermal hyperalgesia and mechanical allodynia.

### Inflammation decreases DRG Car8 expression and increases pITPR1

**Fig 4:**
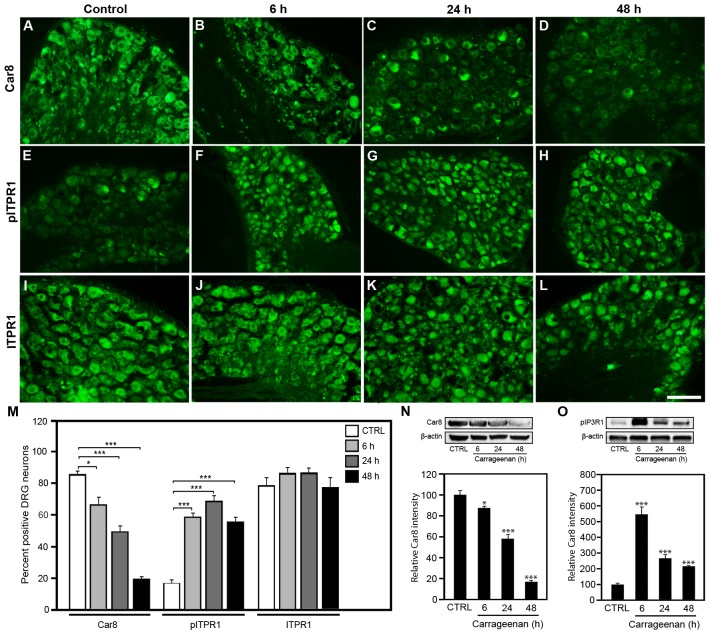
Inflammation decreases DRG Car8 increasing pITPR1. Carrageenan was injected (30 μl 1% carrageenan) into the left hind palm. Analyses of DRG immunohistochemistry of WT rats (Fig. 4A-L); and western blot of rat DRG (Fig. 4N, 4O) show Car8 expression is clearly reduced from 6 h to 48 h (4A-D, M, N) and pITPR1 expression significantly increased from 6h to 48 h (Fig. 4E-H, M, O), while ITPR1 levels remained unchanged (Fig. 4I-M). N = 4. Scale bar: 100 μm. (* P<0.1; ** P<0.01; *** P<0.001; Student’s *t*-test or one-way ANOVA.)

To test whether endogenous Car8 expression changes during inflammation, we injected 30 μl 1% carrageenan in the left hind plantar paw surface of rats, both IHC and western blot analyses of DRG show that neuronal Car8 expression, as a percentage Car8 positive staining of total DRG neurons, is significantly decreased 6 h after carrageenan injections (Fig. 4A-D, M-N), which lasted up to 48 h after injections. In contrast, both IHC and western blots neuronal pITPR1 expression were increased significantly (Fig. 4E-H, M and O) in response to carrageenan, while ITPR1 levels in DRG were unchanged (Fig. 4I-L, M) up to 48 hours after carrageenan. These data suggest that a relative reduction of DRG Car8 expression relative to ITPR1 levels in response to inflammation may be important in the upregulation of pITPR1 and mediating mechanical and thermal hypersensitivity. While the mechanism underlying these responses remains unknown, our data lead us to hypothesize that Car8 is important to inflammatory pain, and that Car8 overexpression may be able to decrease inflammatory pain behaviors.

### Car8 wildtype protein overexpression down regulates ITPR1 phosphorylation and inhibits ATP-induced free calcium release in vitro

**Fig 5:**
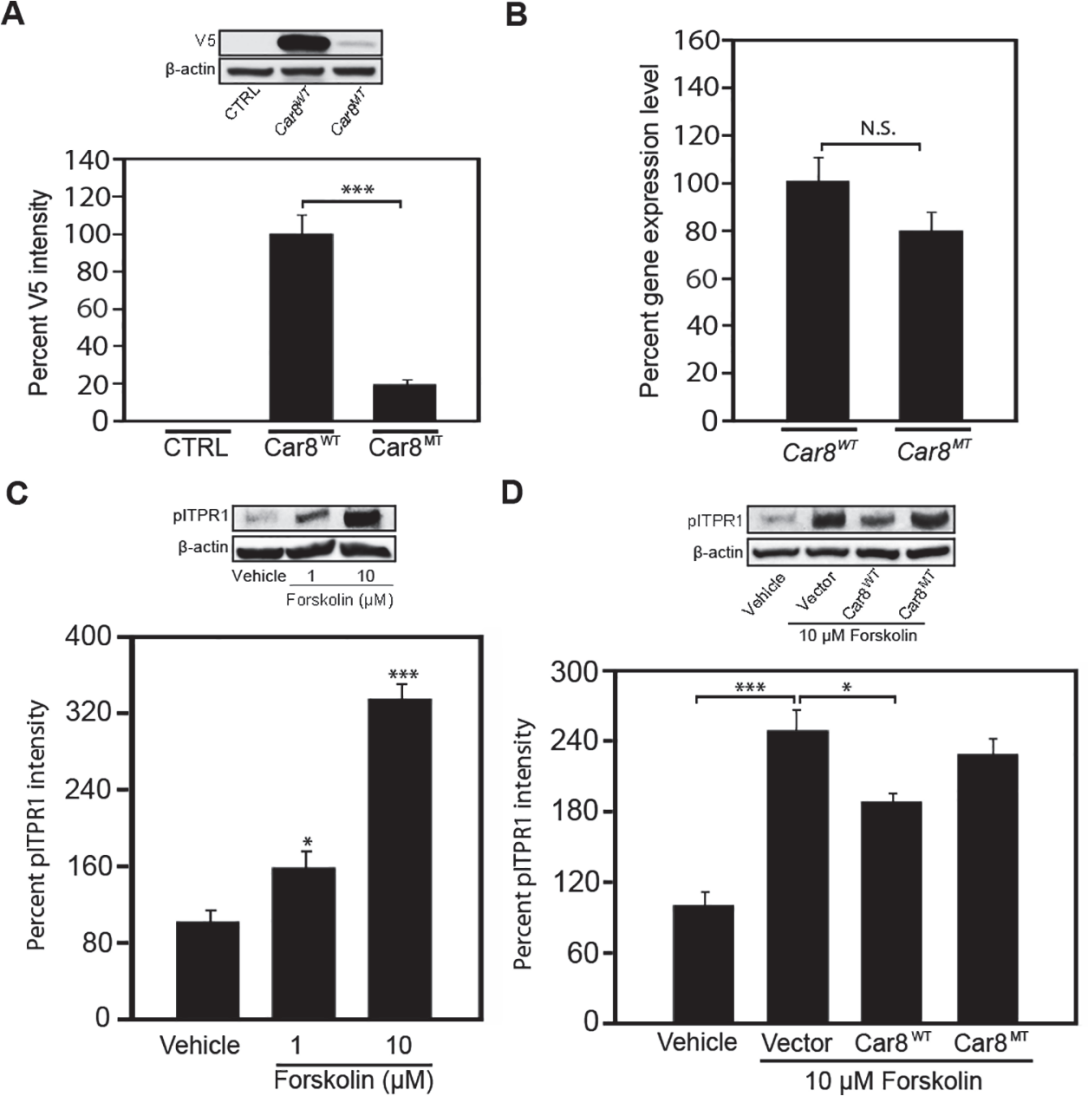
Overexpression of V5-Car8^WT^ *in vitro* inhibits forskolin-induced ITPR1-phosphorylation. Western blotting analyses demonstrate that expression of V5-Car8^WT^ is significantly higher than that of V5-Car8^MT^ in mouse-derived N2A cultures (Fig. 5A). Real-time PCR data show that there is no significant difference between *V5-Car8* ^*WT*^ and *V5-Car8* ^*MT*^ mRNA expression levels from N2A cultures (Fig. 5B). Western blotting analyses of pITPR1 demonstrate that forskolin increases ITPR1 activation intensity in N2A cultures in a dose-dependent manner (Fig. 5C). Overexpression of V5-Car8^WT^ protein using the *AAV2-V5-Car8* ^*WT*^ vector reduced forskolin-induced pITPR1 increases in N2A cultures (Fig. 5D). Overexpression of V5-Car8^MT^ protein using the *AAV2-V5-Car8* ^*WT*^ vector failed to reduce pITPR1 activation by forskolin in N2A cultures (Fig. 5D). N = 6 from 2 independent cultures in triplicate. (* P<0.05; ***P<0.001; Student’s *t*-test or one-way ANOVA.)

To further test whether Car8 regulates ITPR1-dependent cytosolic free calcium, we overexpressed Car8 using gene transfer in mouse N2A cultures. Three adeno-associated virus (AAV) vectors were used in these studies. A control vector *AAV2-eGFP*, which expresses the eGFP gene (Gift from Miami Project Viral Core in University of Miami); and two other vectors that express wildtype Car8 (*AAV2-V5-Car8* ^*WT*^) and the *wdl* mutant Car8 (*AAV2-V5-Car8* ^*MT*^) as a negative control. A *V5* sequence was fused to the C-termini of the *Car8* gene in order to identify the exogenously introduced Car8 protein from endogenously expressed Car8 using an anti-V5 antibody. Using western blot analyses in N2A cultures infected with these AAV constructs, we found Car8^WT^ protein was expressed in N2A cultures at much higher levels vs. the V5-Car8^MT^ protein (Fig. 5A). However, as expected there was no significant difference between *V5*-*Car8* ^*WT*^ and *V5-Car8* ^*MT*^ transcripts using real-time PCR (Fig. 5B). We then tested whether overexpression of Car8^WT^ inhibited forskolin-induced pITPR1. We found forskolin induced the increase of pITPR1 in N2A cultures in a dose-dependent manner (Fig. 5C). The overexpression of V5-Car8^WT^ protein after transfection with the *AAV2-V5-Car8* ^*WT*^ construct inhibited the increase of forskolin-induced pITPR1. In contrast, *AAV2-V5-Car8* ^*MT*^ and *AAV2-eGFP* did not alter pITPR1 levels (Fig. 5D). Our data demonstrate Car8^WT^ is sufficient to inhibit ITPR1 phosphorylation in N2A cells.

**Fig 6:**
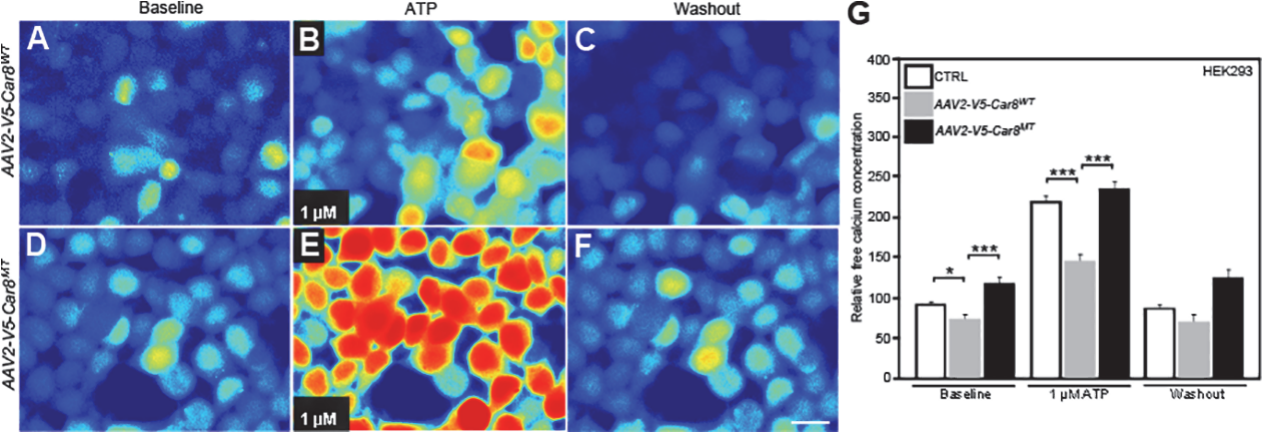
Overexpression of V5-Car8^WT^ *in vitro* inhibits ATP-induced cytoplasmic free calcium increases. Calcium imaging data show overexpression of V5-Car8^WT^ protein reduced 1μM ATP-induced cytoplasmic free calcium increases in HEK293 cultures (Fig. 6A-C, G), when compared to no vector (S1A–F Fig.) and the *V5-Car8* ^*MT*^ vector (Fig. 6D-F, G). (N = 6 from 2 independent cultures in triplicate. * P<0.05; *** P<0.001; one-way ANOVA).

ATP increases ITPR1 dependent calcium release by increasing the open probability of the channel in the presence of activating concentrations of IP3 and calcium [68, 69]. To examine whether Car8 overexpression can inhibit ATP-induced calcium release, we used the *AAV2-V5-Car8* ^*WT*^ and *AAV2-V5-Car8* ^*MT*^ constructs in HEK293 cells and measured real-time intracellular calcium concentrations at baseline and in response to ATP stimulation. ATP stimulated calcium release and an increase in cytosolic free calcium levels in a dose-dependent manner (S1A–F Fig.). We found cytoplasmic free calcium levels after *AAV2-V5-Car8* ^*MT*^ infection were increased at baseline (Fig. 6D, G) and after 1 μM ATP-induced calcium release (Fig. 6E, G) in HEK293 cells. In contrast, free calcium concentrations were reduced relative to control at baseline and after 1 μM ATP after *AAV2-V5-Car8* ^*WT*^ infection (Fig. 6A, B, G). These data demonstrate that Car8 can inhibit ITPR1 activation and thereby reduce baseline and stimulated cytoplasmic free calcium levels.

### Overexpression of Car8 wildtype protein inhibits pITPR1 in DRG of MT mice

Differential AAV-mediated gene transfer to sensory neurons can regulate chronic pain through different routes [70–72]. We next tested for complementation *in vivo* using direct sciatic nerve (SN) injection of our AAV constructs (AAV2 vector packaged in the AAV8 capsid) in Car8 deficient MT mice. To test if Car8 can inhibit ITPR1 phosphorylation in DRG of MT mice, viral particles of *AAV8-V5-Car8* ^*WT*^, *AAV8-V5-Car8* ^*MT*^ and *AAV2-eGFP* were employed using SN injections to express V5-Car8^WT^ (S2A Fig.) and V5-Car8^MT^ (S2C Fig.) proteins in lumbar DRG. SN injections of *AAV8-V5-Car8* ^*WT*^ inhibited ITPR1 phosphorylation in DRG neurons from MT mice (S2A–B, E Fig.); but *AAV8-V5-Car8* ^*MT*^ (S2C–E Fig.) and AAV2-eGFP (data not shown) did not alter pITPR1 levels from baseline despite equal mRNA expression at day 30 after injection (S3 Fig.). Our data demonstrate that *AAV8-V5-Car8* ^*WT*^ overexpression inhibits DRG ITPR1 phosphorylation in MT mice.

We have determined the size distribution (μm^2^) of V5-positive cells in relation to the total V5-positive cell population. V5-positive cells represented 82.1% of cells and these corresponded to mostly small to medium-sized neurons (<700 μm^2^), suggesting *AAV8-V5-Car8* tropism for neurons likely to be involved in nociceptive signaling (Fig. 2E). Immunohistochemistry was next performed to corroborate these findings and further characterize *AAV8-V5-Car8* tropism for putative nociceptive neurons. Lumbar DRG sections were labeled with antibodies against markers of various neuronal populations and assessed for co-localization with V5 (S4 Fig.). The percentage of V5-positive cells that co-localized with calcitonin gene-related peptide (CGRP, S4A–D Fig.), substance P (SP, S4E–H Fig.), or with Isolectin B4 (IB4, S4I–L Fig.), were 62%, 41% and 23%, respectively. As glial cells play a very important role in the mediation of chronic pain [73], we examined whether sciatic nerve delivery resulted in transduction of satellite cells in the DRG and SN. We found the V5 marker was co-localized with S100 in satellite cells (S4M–P Fig., S5E–H Fig.). The pattern of V5 visualization within the spinal cord (SC) recapitulated this transduction of putative nociceptive neurons. Cross sections of the lumbar spinal cord were used in this study. V5-immunoreactive fibers were located mainly within the superficial lamina I and II of the dorsal horn (DH) following sciatic nerve injections, and co-localized with the neuronal marker Tuj1 (S5A–D Fig.). Abundant staining for V5-positive fibers were also observed in sciatic nerve (S5E–H Fig.). Collectively, our data demonstrate robust expression of V5-Car8^WT^ protein in nociceptive neurons after gene transfer and that overexpression of Car8 inhibits pITPR1 and presumably cytosolic free calcium release in DRG of MT mice, as suggested by our *in vitro* data (Fig. 3).

### Overexpression of V5-Car8WT in DRG inhibits hypersensitivity in MT mice

**Fig 7:**
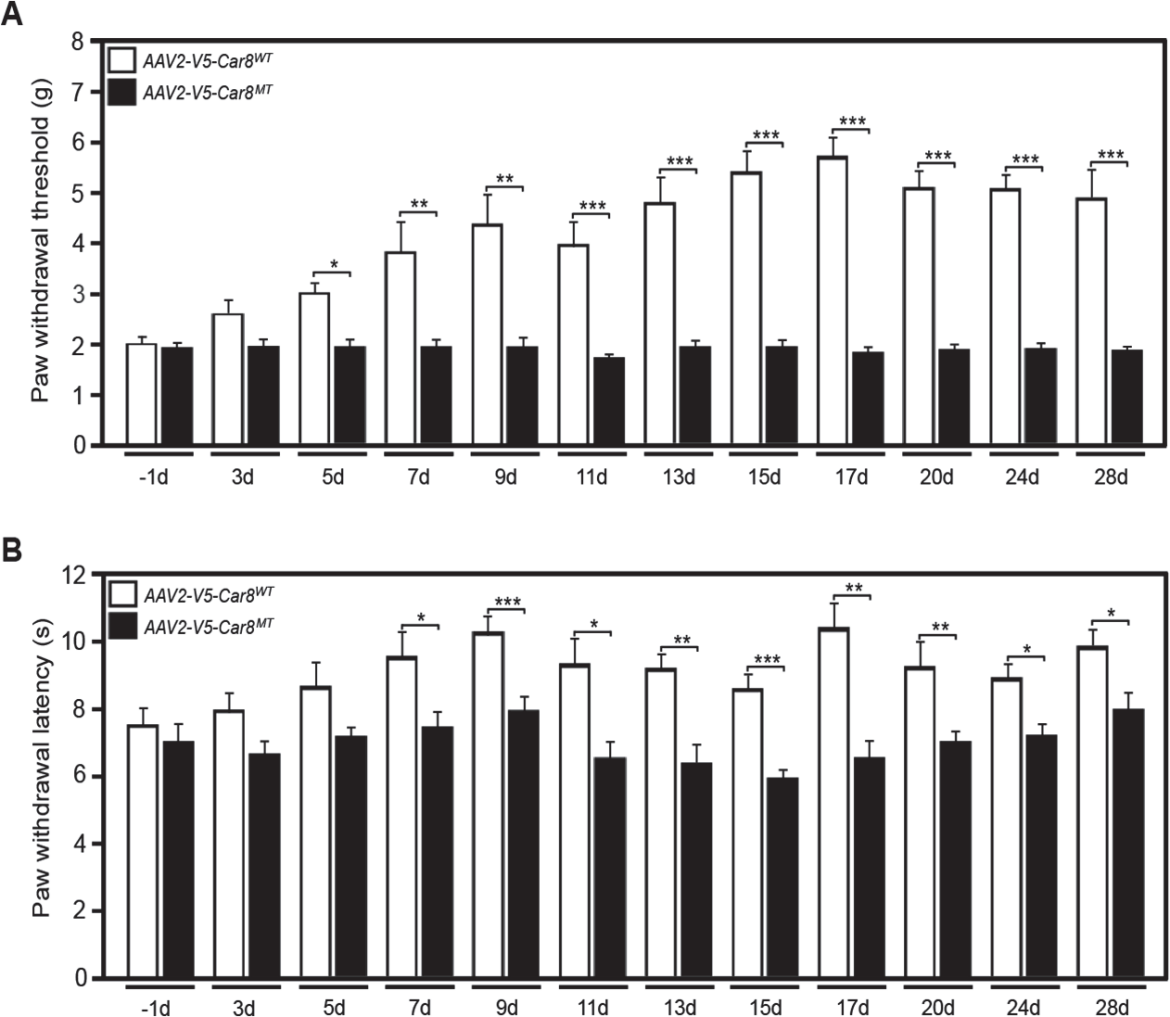
Gene transfer of V5-Car8^WT^ regulates nociception and produces analgesia in MT mice. Sciatic nerve injections of *AAV8-V5-Car8* ^*WT*^ virus (1.5μl, 1.29E+14 genome copies /mL) and *AAV8-V5-Car8* ^*MT*^ virus (1.5μl, 1.61E+14 genome copies /mL) were used in MT mice. (7A) The “up-down” method (see Methods for details) was used to measure mechanical responses by probing the plantar aspect of the hindpaw with von Frey filaments and determining the paw withdrawal threshold (grams). (7B) Thermal withdrawal response latencies were measured to radiant heat (70 units) applied to the plantar aspect of the hind paw (seconds). *AAV8-V5-Car8* ^*WT*^ increased both basal mechanical thresholds (Fig. 7A) and thermal latencies (Fig. 7B), starting on day 7 after injection and lasting more than 28 days. Sciatic nerve injections of *AAV8-V5-Car8* ^*MT*^ failed to affect nociception (Fig. 7A, B) over the 28 d. (N = 8. * P<0.1; ** P<0.01; *** P< 0.001; Student *t*-test and two-way repeated measure ANOVA.)

We have previously shown that SN injections of *AAV8-V5-Car8* ^*WT*^ overexpressed V5-Car8^WT^ protein and inhibited ITPR1 activation in DRG neurons from MT mice (S2A–B, E Fig.). We next tested if this complementation experiment using SN gene transfer was necessary and sufficient to correct baseline mechanical and thermal pain related behaviors in MT mice. We found that mechanical withdrawal threshold and thermal withdrawal latencies were significantly improved approximately 5 and 7 days after sciatic nerve injection with *AAV8-V5-Car8* ^*WT*^, and the effect lasted more than 28 days (Fig. 7A-B). The *AAV8-V5-Car8* ^*MT*^ (negative control)(Fig. 7A-B) and *AAV2-eGFP* vectors (data not shown) failed to show any improvement in pain behaviors. These *in vivo* complementation data demonstrate that Car8 overexpression is able to reverse the pain behaviors observed in MT mice.

### Gene transfer of Car8WT treats inflammatory pain

**Fig 8:**
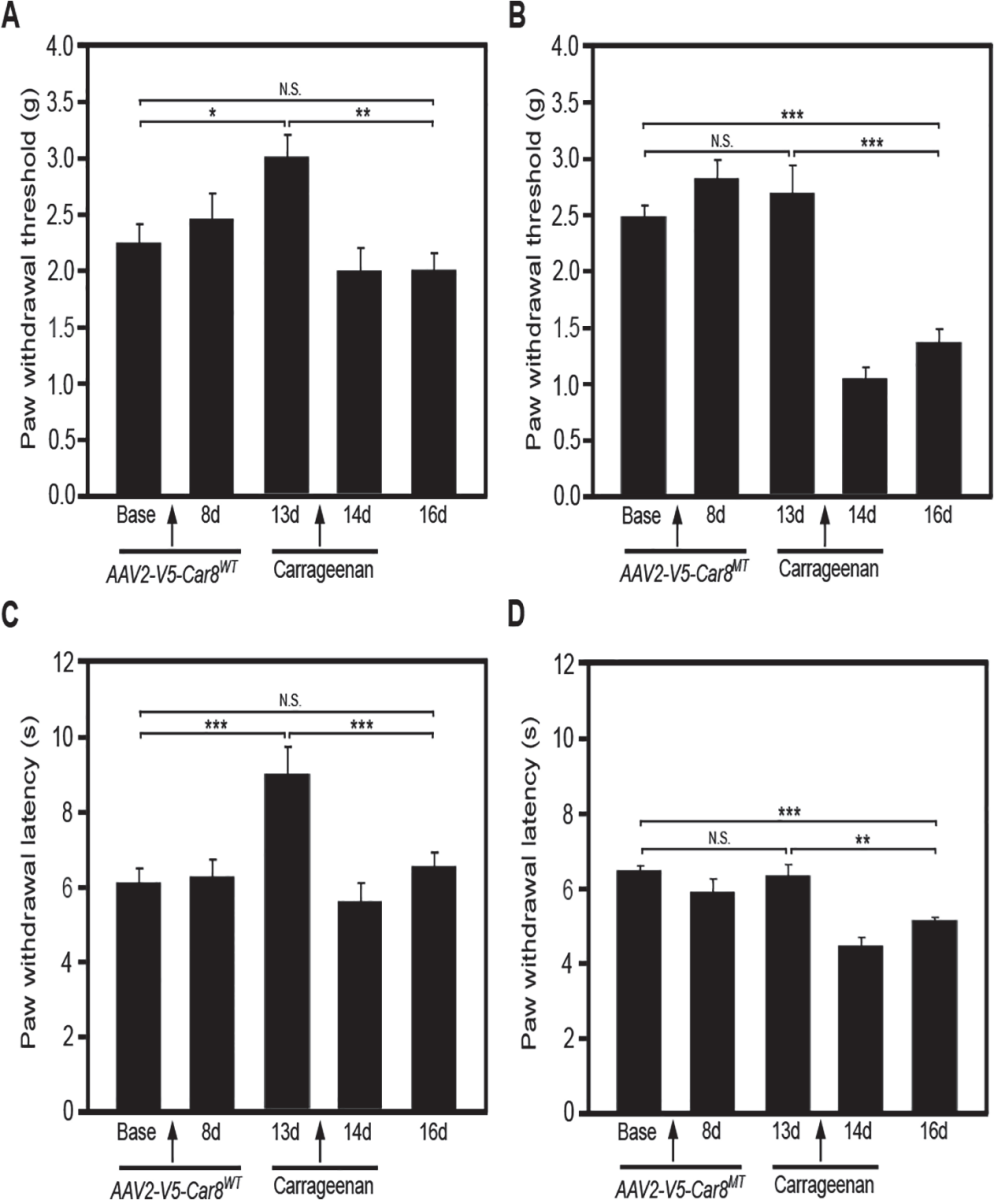
Gene transfer of V5-Car8^WT^ produces analgesia and anti-hyperalgesia in a carrageenan subacute inflammatory pain model in WT mice. Sciatic nerve injections of *AAV8-V5-Car8* ^*WT*^ virus (1.5μl, 1.29E+14 genome copies /mL) increase basal mechanical thresholds (Fig. 8A) and thermal latencies (Fig. 8C) by day 13, before carrageenan injection. Sciatic nerve injections of *AAV8-V5-Car8* ^*MT*^ virus (1.5μl, 1.61E+14 genome copies /mL) failed to alter basal mechanical thresholds (Fig. 8B) and thermal latencies (Fig. 8D) by day 13, before carrageenan injection. After carrageenan injections, the *AAV8-V5-Car8* ^*WT*^ virus group showed a reduction in mechanical thresholds (Fig. 8A) and thermal latencies (Fig. 8C) on days 14 and 16 when compared to day 13; but these did not differ from baseline. After carrageenan injections, the *AAV8-V5-Car8* ^*MT*^ virus group showed a reduction in mechanical thresholds (Fig. 8B) and thermal latencies (Fig. 8D) well below day 13 and baseline. (N = 8. * P<0.1; ** P<0.01; *** P<0.001 by one-way ANOVA.)

We next tested if DRG overexpression of V5-Car8^WT^ could reverse carrageenan-induced hyperalgesia in WT mice. Once again, we delivered *AAV8-V5-Car8* ^*WT*^ and *AAV8-V5-Car8* ^*MT*^ (as a negative control) to DRG using direct sciatic nerve injections in WT mice and then delivered carrageenan to the same side hind paw palm 13 days after SN injections. We found that SN delivery of *AAV8-V5-Car8* ^*WT*^ produced *analgesia* as demonstrated by a significant increase mechanical and thermal baseline responses, peaking on day 13. Carrageenan delivery after behavior data collection on day 13 clearly reduced mechanical (Fig. 8A) and thermal (Fig. 8C) baseline responses on day 14 and through day 16, as compared with day 13 responses. Importantly, there were no significant differences between these responses when compared with that on day zero (Fig. 8A, C). In contrast, SN *AAV8-V5-Car8* ^*MT*^ injections failed to increase the thermal and mechanical baseline response in WT mice (Fig. 8B, D). The delivery of carrageenan on day 13 once again induced mechanical and thermal hyperalgesia on days 14 through day 16 (Fig. 8B, D), but these responses were lower when compared to baseline. These data demonstrate that *Car8* ^*WT*^ gene transfer produces both analgesic and anti-hyperalgesic effects in this inflammatory model.

**Fig 9:**
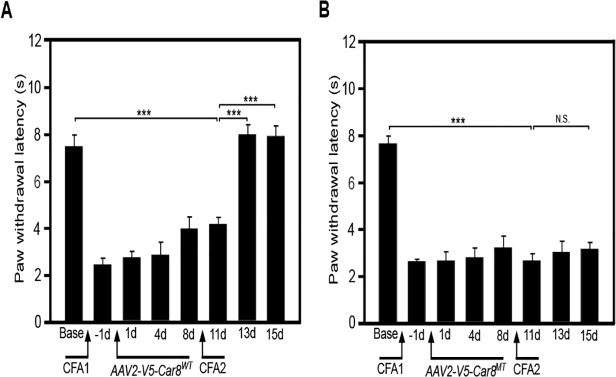
Gene transfer of V5-Car8^WT^ produces thermal anti-hyperalgesia in a complete Freund’s adjuvant (CFA) chronic inflammatory pain model in WT mice. Chronic inflammatory pain produced by injection of 30 μl 1% CFA in the left hind palm on minus day 2, and day 9. CFA induces thermal hyperalgesia starting on minus d1. Sciatic nerve injections of *AAV8-V5-Car8* ^*WT*^ virus (1.5μl, 1.29E+14 genome copies /mL) on day zero increased basal latencies (Fig. 9A) by day 13. In contrast, sciatic nerve injections of *AAV8-V5-Car8* ^*MT*^ virus (1.5μl, 1.61E+14 genome copies /mL) failed to alter basal thermal latencies (Fig. 9B) at any time. (N = 8. *** P<0.001 by one-way ANOVA.)

Finally, to further study whether SN delivery of *AAV8-V5-Car8* ^*WT*^ inhibits chronic inflammatory pain, we tested the effects of *AAV8-V5-Car8* ^*WT*^ and *AAV8-V5-Car8* ^*MT*^ gene transfer on CFA-induced inflammatory pain. CFA was injected into the same side hind palm on minus day 1 and day 9, after sciatic nerve injection of *AAV8-V5-Car8* ^*WT*^ or *AAV8-V5-Car8* ^*MT*^. We found V5-Car8^WT^ expressing constructs, but not the V5-Car8^MT^ expressing constructs, significantly inhibited CFA-induced thermal hyperalgesia on day 13 (Fig. 9A, B). Collectively, these *in vivo* findings suggest that Car8 modulates nociceptor hypersensitivity and susceptibility to the acute-to-chronic pain transition in this model of chronic inflammatory pain *in vivo*.

## Discussion

### The biologic significance of Car8 deficiency

Cytoplasmic free calcium concentrations regulate many critical cellular functions including neuronal excitability, neurite outgrowth, neurotransmitter release, mitochondrial energy production, apoptosis, and neuroplasticity associated with long-lasting adaptive responses [74–80]. Any imbalance in these inputs associated with cytoplasmic free calcium dysregulation are likely to affect pain processing in the central or peripheral nervous system leading to altered pain thresholds, the summation of heat pain, and pain perception [81, 82]. Our studies demonstrate that Car8 plays an important role in a variety of physiologic processes clinically relevant to chronic inflammatory pain. We found that a deficiency of Car8 either at baseline (genetic variation affecting protein stability and expression as demonstrated in this SCA model)(see Fig. 1), or a Car8 deficiency relative to ITPR1 resulting from inflammation (see Fig. 4), enhances DRG pITPR1, causes increases in cytoplasmic free calcium concentrations, and promotes inflammatory pain behaviors (Figs. 7–9).

### Car8 inhibits inflammatory pain by reducing ITPR1-dependent cytosolic free calcium release

With regard to the mechanism of hypersensitivity associated with Car8 deficiency in this SCA model, our data demonstrate that increased steady-state pITPR1 and increases in cytosolic free calcium are required. Using MT mice, and sciatic nerve gene transfer (*AAV8-V5-Car8*), we showed that Car8 complementation abrogated (1) the increased steady-state pITPR1, (2) increases in steady-state cytosolic free calcium, (3) stimulated intracellular calcium release (Figs. 3 and 6), and (4) baseline hypersensitivity. Moreover, we were able to show in WT animals that Car8 overexpression was able to produce both analgesia and anti-hyperalgesia in complex pain behaviors associated with carrageenan and CFA inflammation (Figs. 8 and 9). Collectively, these findings indicate that environmental and genetic changes in Car8 expression alter ITPR1 regulation and its control of cytoplasmic free calcium homeostasis, provoking the acute-to-chronic pain transition in our inflammatory models. Long-term potentiation associated with chronic inflammatory pain disorders results from cytoplasmic free calcium dysregulation [4–9]. Our data provide strong evidence that genetic or epigenetic alterations in Car8 expression, or function, may have major consequences on cytoplasmic free calcium regulation. Moreover, ITPR1-mediated calcium release is known to transduce signals from metabotropic receptor activation stimulating cellular entry of calcium through voltage- and receptor-gated calcium channels (such as N-methyl-D-aspartate receptors) [13, 14, 16] implicated in chronic pain behaviors [16] and other neurodegenerative disorders [1]. Our data support a pivotal role for Car8 in its regulation of ITPR1 and intracellular calcium levels, shown to have far reaching effects on nociceptive activity, inflammatory pain behaviors, and the development of long-term hypersensitivity.

### The intersection between Car8, ITPR1, cytosolic free calcium, inflammatory pain and other forms of neuropathology

Calcium signals are specifically thought to couple synaptic activity to gene regulation, thereby serving as the critical messenger between synapses and the nucleus [11, 48, 74, 77, 83, 84]. Intracellular calcium homeostasis therefore is tightly regulated by an assortment of G protein-coupled receptors, ion channels, calcium binding proteins, transcriptional networks, and ion exchangers [37, 39]. Calcium dysregulation and neuronal dysfunction are implicated in numerous other inflammatory processes including ischemic stroke, multiple sclerosis, Alzheimer's disease, Huntington's disease and epilepsy [85–89]. In addition to Car8, ITPR1 function is impacted by a family of regulatory proteins including presenilin, huntingtin, DANGER, 80K-H, and cytochrome C. Mutations in two presenilin genes (PS1, PS2) account for the majority of familial early-onset Alzheimer's disease [89]. Evidence from a number of studies now indicate that PS1 and PS2 mutations alter the presynaptic regulation of intracellular calcium signaling pathways, suggesting that these disturbances may represent a common early pathogenic mechanism of presenilin-associated familial Alzheimer’s disease [1, 88, 89]. Based on our findings, it will be important to define how chronic inflammation associated with various neurodegenerative disorders impacts Car8 expression and ITPR1 function.

## Conclusions

Our data demonstrate that Car8 may represent a ‘molecular linchpin’ critical to pain perception, pain behaviors, synaptic plasticity, including long-term potentiation and persistent inflammatory pain by regulating ITPR1 and cytosolic free calcium. Moreover, these findings may have important implications for the future in a number of common chronic disorders where inflammation-induced Car8 deficiency may play an important role in disruption of calcium homeostasis, persistent pain and disease progression. These studies indicate that Car8 regulation of ITPR1 and cytoplasmic free calcium homeostasis during the acute-to-chronic pain transition may be of critical importance for the development of novel therapeutic strategies. Finally, our work provides the first preclinical therapeutic rationale for using Car8 as an allosteric inhibitor of ITPR1 to treat chronic inflammatory pain.

## Supporting Information

S1 Fig Overexpression of V5-Car8^WT^ in vitro inhibits ATP-induced cytoplasmic free calcium increases. Calcium imaging data show ATP-induced cytoplasmic free calcium increases in a dose-response manner in HEK293 cultures (S1A–F Fig.). (N = 6 from 2 independent cultures in triplicate. * P<0.05; *** P<0.001; one-way ANOVA).
(TIF)

S2 Fig Overexpression of V5-Car8WT after gene transfer via sciatic nerve injection inhibits ITPR1-activation. Overexpression of V5-Car8^WT^ protein in DRG of MT mice using the AAV8-V5-Car8^WT^ vector reduces pITPR1 levels at day 30 after sciatic nerve injection (S2B, E Fig.) when compared to overexpression of V5-Car8^MT^ protein using the AAV8-V5-Car8^MT^ vector at the same time point (S2D–E Fig.). (N = 4. *** P<0.001; Student t-test.)
(TIF)

S3 Fig RealTime-PCR of exogenous Car8 30 days after sciatic nerve injection of AAV8-V5-Car8^MT^ and AAV8-V5-Car8^WT^ vectors. There was no significant difference in the expression of these vectors as demonstrated by steady-state mRNA levels of V5-Car8 in DRG tissues (S3 Fig.). Contralateral DRGs (data not shown) were used as a negative control. (N = 5. P = 0.823 by Student t-test)
(TIF)

S4 Fig Tropism of AAV8-V5-Car8 constructs in DRG 30 days after sciatic nerve injections. Immunoreactivity for anti-V5 (S4A, E, I, M Fig.) together with anti-calcitonin gene-related peptide (CGRP) (S4B–D Fig., red), anti-substance P (SP) (S4F–H Fig., red), anti-isolectin B4 (IB4) (S4J–L Fig., red) and anti-S100 (S4N–P Fig., red) antibodies, respectively. Panels C, G, K and O are merged images from A and B, E and F, I and J, M and N, respectively. D, H, L and P are high amplification images of framed areas in C, G, K and O, respectively. Arrows (A-P) demonstrates that V5-Car8 co-localizes with CGRP, SP and IB4 in DRG neurons as well as with S100 in satellite cells after sciatic nerve injections of AAV8-V5-Car8^WT^. (N = 5. Scale bar = 50 μm.)
(TIF)

S5 Fig Expression of V5-Car8^WT^ in the spinal cord (SC) and the sciatic nerve (SN). V5-Car8^WT^ in SC and the SN were measured 30 days after injection of AAV8-V5-Car8^WT^ virus into sciatic nerves of MT mice (S5A–H Fig.). Immunoreactivity for anti-V5 (S5A, E Fig., green), together with anti-Tuj1 (S5B Fig., red), and anti-S100 antibodies, respectively (S5F Fig., red). Longitudinal SC (S5A–D Fig.) and SN (S5E–H Fig.) images were taken from the ipsilateral MT mice at the level of L4–5 and S1 spinal cord and ipsilateral SN, respectively. Images S5C and G are merged from S5A and B; and S5E and F, respectively. Images S5D and H are high amplifications of framed areas in S5C and G, respectively. (N = 4–5. Scale bar = 50 μm)
(TIF)
