# Supplementary figures and images for: Carbonic Anhydrase-8 Regulates Inflammatory Pain by Inhibiting the ITPR1-Cytosolic Free Calcium Pathway
Source: PLoS One. 2015 Mar 3;10(3):e0118273. doi: 10.1371/journal.pone.0118273 (PMC4347988; doi:10.1371/journal.pone.0118273)

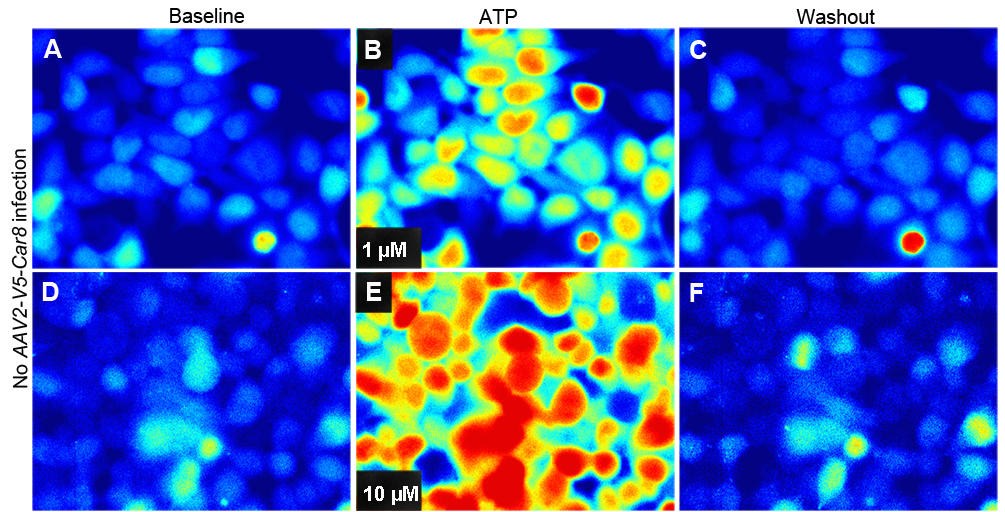

Supplement: S1 Fig — Calcium imaging data show ATP-induced cytoplasmic free calcium increases in a dose-response manner in HEK293 cultures (S1A–F Fig.). (N = 6 from 2 independent cultures in triplicate. * P<0.05; *** P<0.001; one-way ANOVA). (TIF) [file pone.0118273.s001.tif]

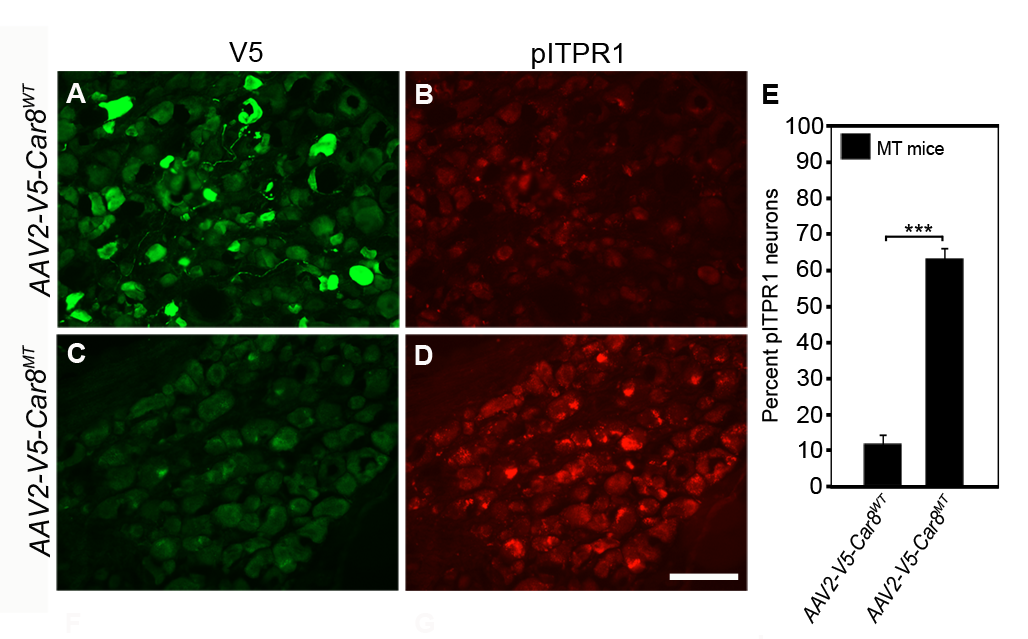

Supplement: S2 Fig — Overexpression of V5-Car8WT protein in DRG of MT mice using the AAV8-V5-Car8WT vector reduces pITPR1 levels at day 30 after sciatic nerve injection (S2B, E Fig.) when compared to overexpression of V5-Car8MT protein using the AAV8-V5-Car8MT vector at the same time point (S2D–E Fig.). (N = 4. *** P<0.001; Student t-test.) (TIF) [file pone.0118273.s002.tif]

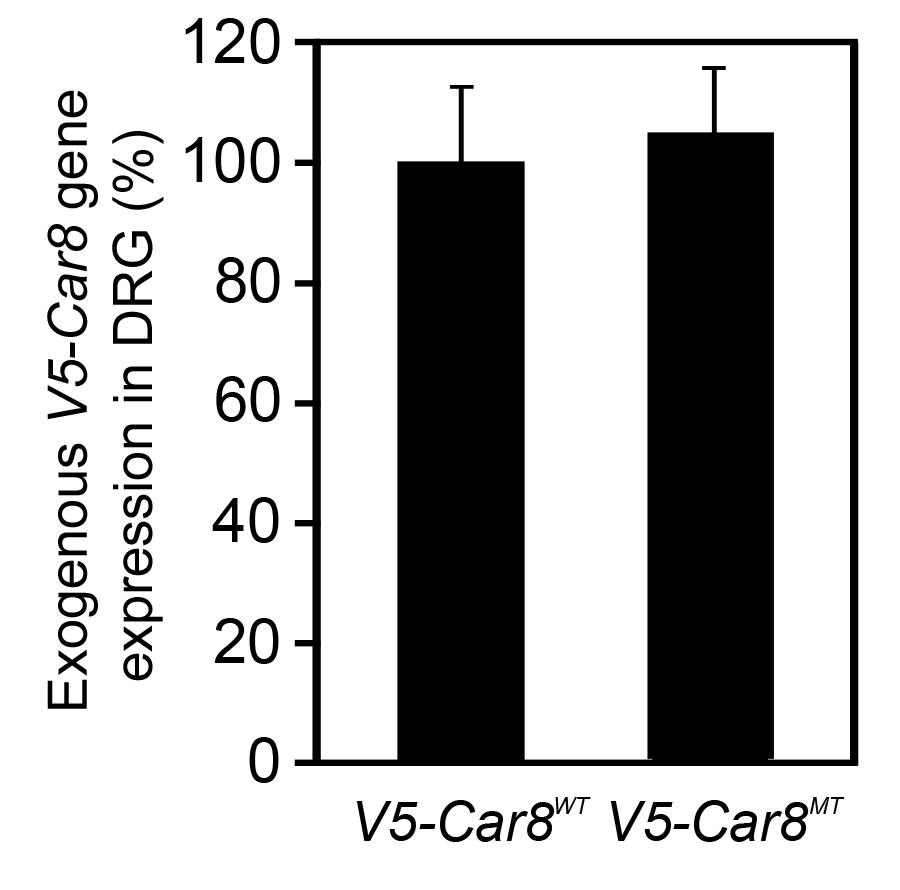

Supplement: S3 Fig — There was no significant difference in the expression of these vectors as demonstrated by steady-state mRNA levels of V5-Car8 in DRG tissues (S3 Fig.). Contralateral DRGs (data not shown) were used as a negative control. (N = 5. P = 0.823 by Student t-test) (TIF) [file pone.0118273.s003.tif]

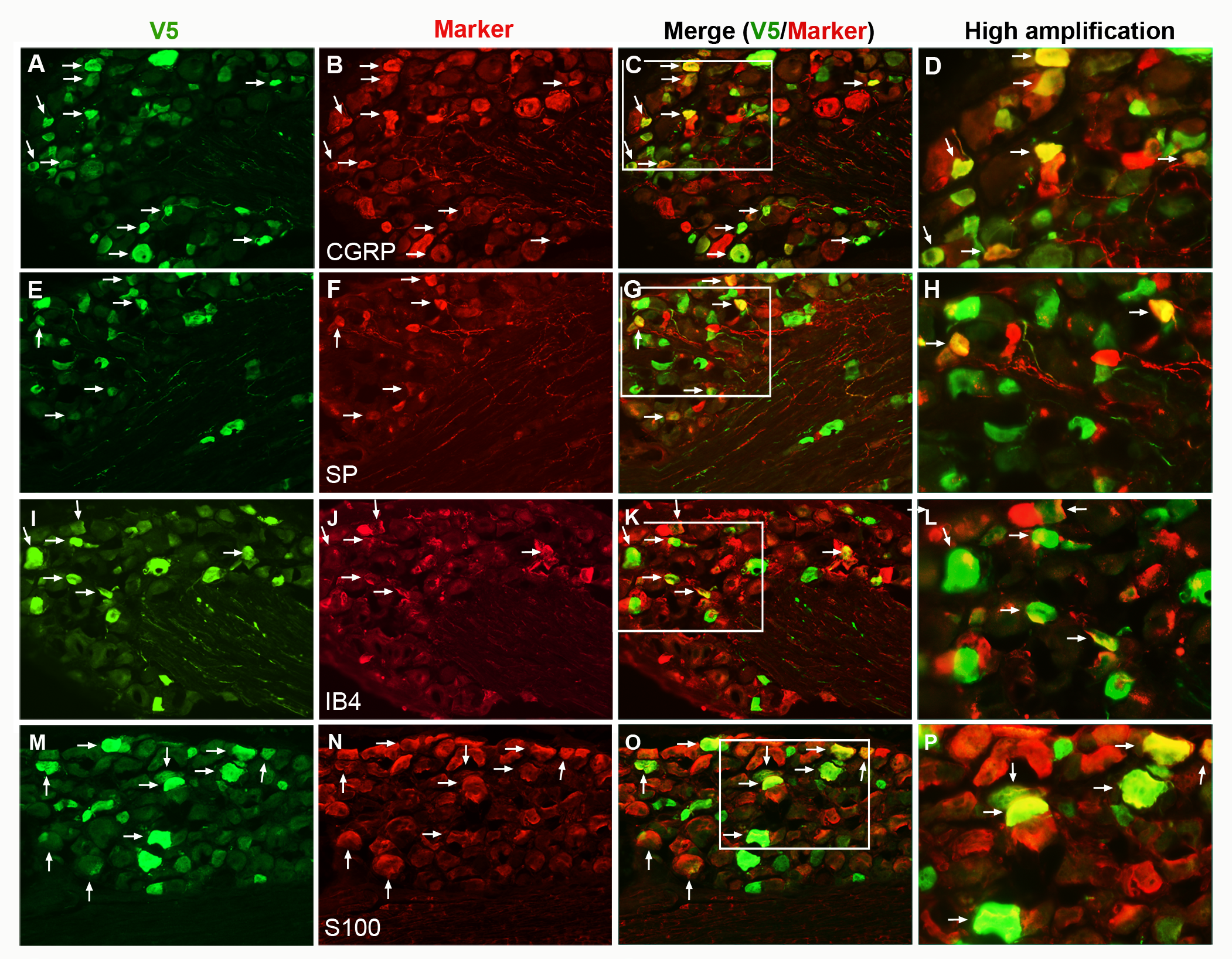

Supplement: S4 Fig — Immunoreactivity for anti-V5 (S4A, E, I, M Fig.) together with anti-calcitonin gene-related peptide (CGRP) (S4B–D Fig., red), anti-substance P (SP) (S4F–H Fig., red), anti-isolectin B4 (IB4) (S4J–L Fig., red) and anti-S100 (S4N–P Fig., red) antibodies, respectively. Panels C, G, K and O are merged images from A and B, E and F, I and J, M and N, respectively. D, H, L and P are high amplification images of framed areas in C, G, K and O, respectively. Arrows (A-P) demonstrates that V5-Car8 co-localizes with CGRP, SP and IB4 in DRG neurons as well as with S100 in satellite cells after sciatic nerve injections of AAV8-V5-Car8WT. (N = 5. Scale bar = 50 μm.) (TIF) [file pone.0118273.s004.tif]

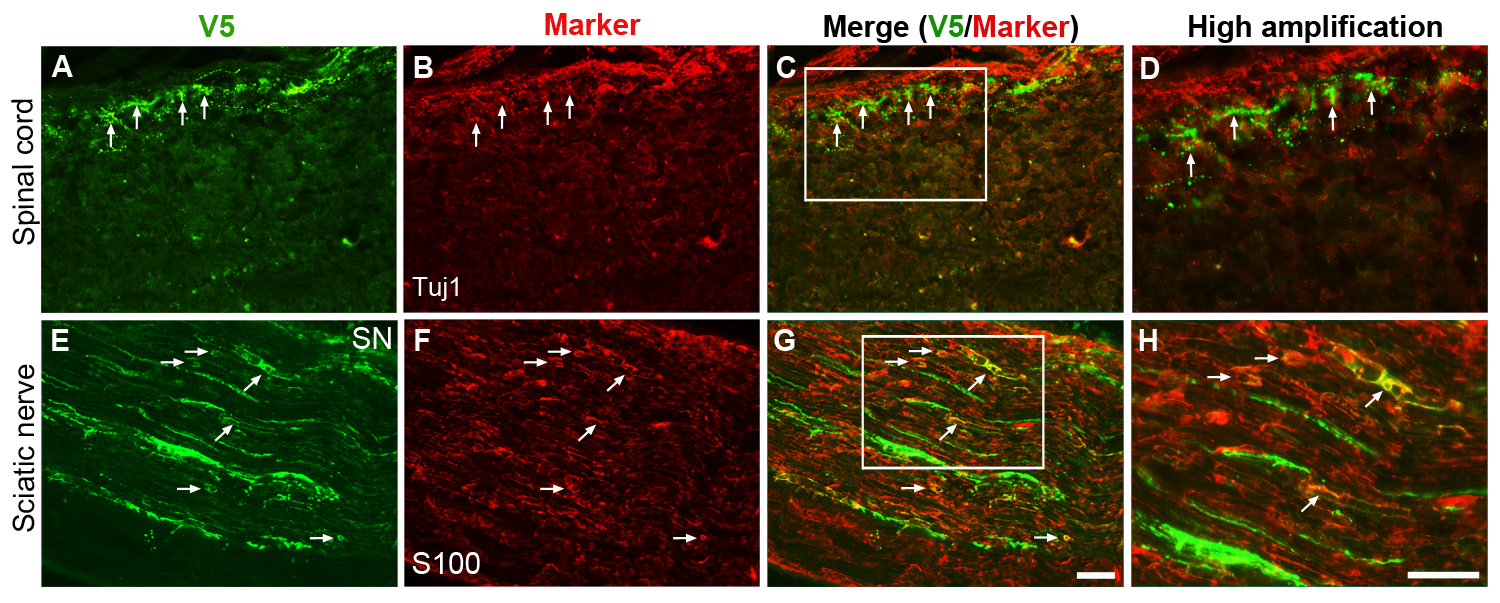

Supplement: S5 Fig — V5-Car8WT in SC and the SN were measured 30 days after injection of AAV8-V5-Car8WT virus into sciatic nerves of MT mice (S5A–H Fig.). Immunoreactivity for anti-V5 (S5A, E Fig., green), together with anti-Tuj1 (S5B Fig., red), and anti-S100 antibodies, respectively (S5F Fig., red). Longitudinal SC (S5A–D Fig.) and SN (S5E–H Fig.) images were taken from the ipsilateral MT mice at the level of L4–5 and S1 spinal cord and ipsilateral SN, respectively. Images S5C and G are merged from S5A and B; and S5E and F, respectively. Images S5D and H are high amplifications of framed areas in S5C and G, respectively. (N = 4–5. Scale bar = 50 μm) (TIF) [file pone.0118273.s005.tif]
